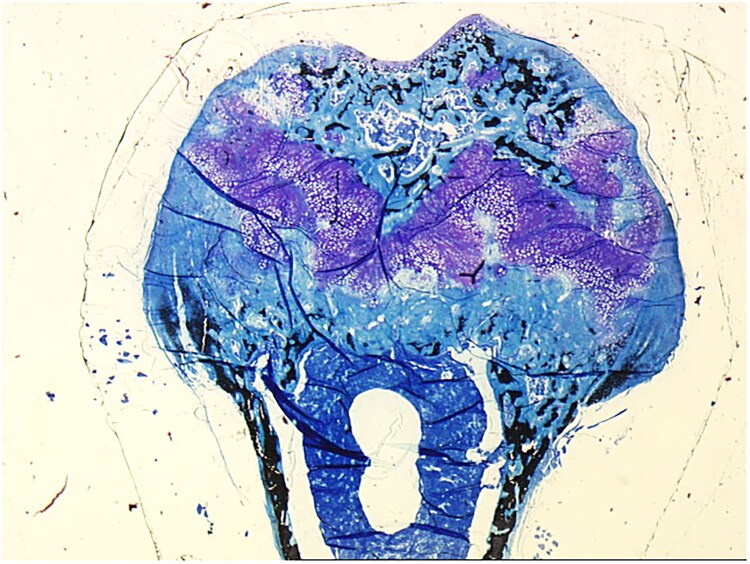# Correction to: “Transgenic Mice Overexpressing Human Fibroblast Growth Factor 23 (R176Q) Delineate a Putative Role for Parathyroid Hormone in Renal Phosphate Wasting Disorders”

**DOI:** 10.1210/endocr/bqag058

**Published:** 2026-05-18

In above-named article by Bai X, Miao D, Li J, Goltzman D, and Karaplis AC (*Endocrinology*, 2004, 145(11): 5269-5279; doi: 10.1210/en.2004-0233), there was an error in Figure 3C.

In the original article, in Figure 3, panel C, a concern has been raised regarding the presence of repetitive patterns in the histological image. Upon review, the authors have confirmed that during preparation of this figure, limited cosmetic modifications were made to regions of the bone marrow background by duplicating small image areas to improve visual appearance in locations affected by tissue-processing artifacts. These modifications were not disclosed in the original publication and do not comply with current image presentation standards.

According to the authors, the modifications were confined to background marrow regions and did not involve mineralized or nonmineralized trabecular bone, nor did they affect the data or conclusions reported in the study. The corrected, unprocessed image corresponding to Figure 3C has now been provided below.

**Original Figure 3:**

**Figure:**
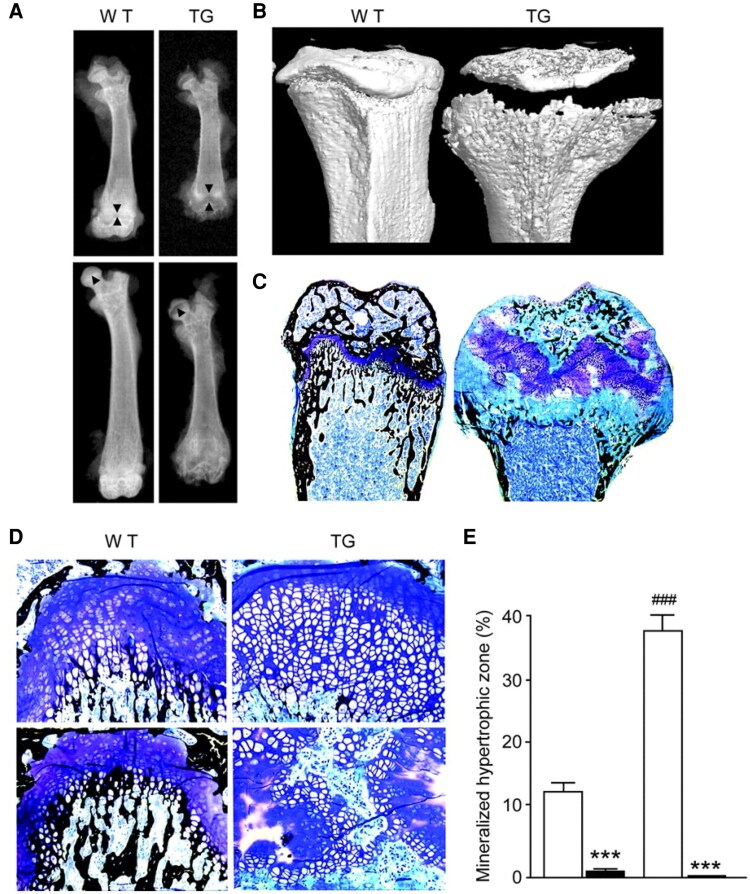


**Corrected Figure 3C:** doi: https://doi.org/10.1210/en.2004-0233

**Figure:**